# Variation of Proteolytic Cleavage Sites towards the N-Terminal End of the S2 Subunit of the Novel SARS-CoV-2 Omicron Sublineage BA.2.12.1

**DOI:** 10.3390/molecules27185817

**Published:** 2022-09-08

**Authors:** Nadine Anna Schilling, Hubert Kalbacher, Timo Burster

**Affiliations:** 1 Faculty of Organic Chemistry, Eberhard Karls University Tübingen, 72076 Tübingen, Germany; 2 Institute of Clinical Anatomy and Cell Analysis, Faculty of Medicine, Eberhard Karls University Tübingen, Österbergstraße 3, 72074 Tübingen, Germany; 3 Department of Biology, School of Sciences and Humanities, Nazarbayev University, Kabanbay Batyr Ave. 53, Nur-Sultan 010000, Kazakhstan

**Keywords:** neutrophil elastase, proteinase 3, neutrophils, serine proteases, COVID-19, SARS-CoV-2, Omicron variant, cathepsin G, BA.2.12.1

## Abstract

The prevalence of novel SARS-CoV-2 variants is also accompanied by an increased turnover rate and additional cleavage sites at the positions necessary for priming the Spike (S) protein. Of these priming sites, the proteolytically sensitive polybasic sequence of the activation loop at the S1/S2 interface and the S2′ location within the S2 subunit of the S protein are cleaved by furin and TMPRSS2, which are important for the infection of the target cell. Neutrophils, migrating to the site of infection, secrete serine proteases to fight against pathogens. The serine proteases encompass neutrophil elastase (NE), proteinase 3 (PR3), and cathepsin G (CatG), which can hydrolyze the peptide bond adjacent to the S1/S2 interface. SARS-CoV-2 might take the opportunity to hijack proteases from an immune response to support viral entry to the cell. The region near S704L within the S2 subunit, a novel amino acid substitution of SARS-CoV-2 Omicron sublineage BA.2.12.1, is located close to the S1/S2 interface. We found that NE, PR3, and CatG digested the peptide within this region; however, the S704L amino acid substitution altered cleavage sites for PR3. In conclusion, such an amino acid substitution modifies S2 antigen processing and might further impact the major histocompatibility complex (MHC) binding and T cell activation.

## 1. Introduction

The continuous mutation of the Spike (S) protein of the severe acute respiratory syndrome coronavirus 2 (SARS-CoV-2) is an immune evasion strategy of the virus. One of the immune evasion strategies encompasses the escape from extracellular immune detection by evasion into the target cell. Several mechanisms account for an increased infection rate, including the enhanced receptor binding and proteolytic cleavage of the S protein at the S1/S2 interface and S2′ site internal to the S2 subunit [1]. The prevalence of novel proteolytic cleavage sites during the progression of amino acid substitutions within the polybasic sequence of the S1/S2 interface of SARS-CoV-2 variants might promote this process even further [2,3]. The cleavage of the polybasic sequence (RRAR↑S) primarily by furin is a prerequisite for further hydrolysis of the S2′ site (PSKR↑S) to initiate the membrane-fusion process. Interestingly, the S1/S2 interface is already hydrolyzed by furin; however, the remaining S1 subunit, required to attach to the angiotensin-converting enzyme 2 (ACE2), non-covalently associates with the S2 subunit in the infected producer cell before the virions are released. In comparison, the S2′ site needs to be processed by the host protease, predominantly by cell surface transmembrane protease serine subtype 2 (TMPRSS2) via cell surface entry or by cathepsin L following the clathrin-mediated endocytosis pathway (TMPRSS2 independent) to generate the fusion peptide. In both pathways, the fusion peptide, more precisely the heptad repeat 1, propels the fusion peptide towards the membrane initiating the fusion pore so that the viral genome can be released into the cytosol [4,5].

In particular, the appearance of novel amino acid substitutions within the S protein changes the protease-mediated SARS-CoV-2 entrance of the host cell, including viral transmission, immune escape, and increased pathogenicity [6,7]. The distribution of the SARS-CoV-2 Omicron sublineage, referred to as BA.2.12.1, is rapidly growing in several regions of the world. BA.2.12.1 comprises additional amino acid substitutions at position 452 (L → Q, L452Q), having a similar binding capacity to ACE2 receptor like BA.2 [8], and at S704L, which is close to the polybasic sequence (https://covariants.org/variants/22C.Omicron, accessed on 18 July 2022, [9]). Recent data showed that the neutralizing antibody titer was lower by a factor of 14.1 against BA.2.12.1 (6.4 against BA.1, 7.0 against BA.2, and 21.0 against both BA.4 or BA.5) in comparison to the control (WA1/2020) [10], and the L452Q mutation might be responsible for humoral immune evasion [8], which indicates the need to develop vaccines provoking neutralizing antibodies towards the more conserved S2′ region [11].

Activated neutrophils exocytose neutrophil serine proteases (NSPs), such as neutrophil elastase (NE), proteinase 3 (PR3), cathepsin G (CatG), and neutrophil serine protease 4 (NSP4), at the site of infection [12]. In the case of SARS-CoV-2 infection, high levels of CatG and NE are present in nasopharyngeal swabs of patients with SARS-CoV-2 [13], and high CatG was found in plasma from patients depending on their COVID-19 status [14]. Whether the novel amino acid substitution of S704L might alter the proteolytic cleavage signature of the S protein is unclear. To this end, we synthesized peptides spanning the S704L sequence, examined the cleavage pattern after incubation with CatG, NE, and PR3, and found that the S704L mutation adjusted the cleavage sites for PR3.

## 2. Results and Discussion

**Figure 1:**
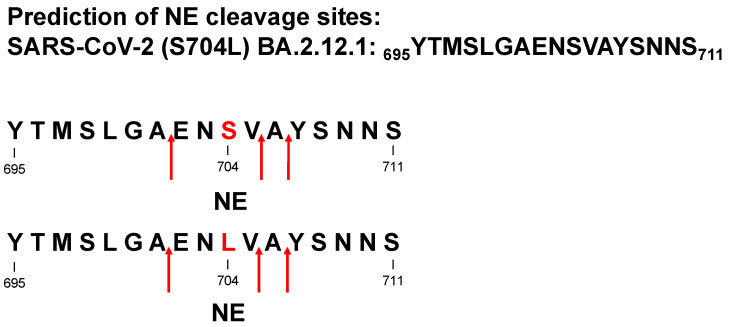
Cleavage site prediction for NE. Potential cleavage sites of _695_YTMSLGAEN**S**VAYSNNS_711_ (S704 peptide) and _695_YTMSLGAEN**L**VAYSNNS_711_ (L704 peptide) were mapped for NE by using “ExPASy peptide cutter” and indicated by red arrows.

**Figure 2:**
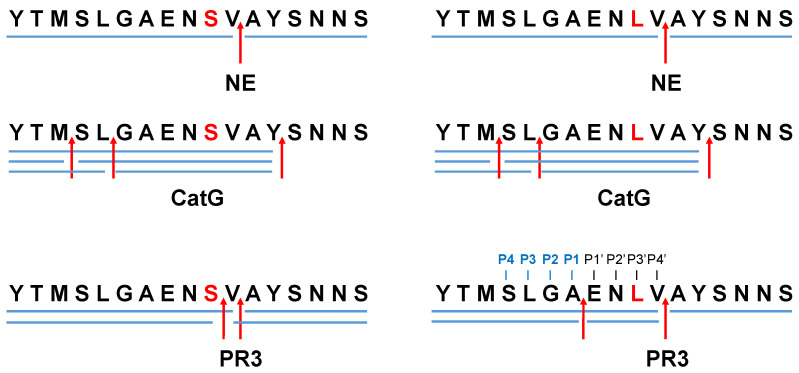
Proteolytic cleavage sites for NE, CatG, and PR3. S704 and L704 peptides (200 μg/mL) were incubated with 4 μg/mL of NE, CatG, or PR3 for 2 h at 37 °C. The hydrolysis of the peptide bonds by the indicated proteases is summarized in a digestion map, where blue bars denote the digestion pattern and red arrows the cleavage sites. Four independent experiments for HPLC (n = 4), MALDI-TOF-MS (n = 2), and HPLC-ESI-QTOF-HRMS (n = 2) were conducted.

Protease-catalyzed hydrolysis ensues specifically between the P1 and P1′ peptide bond. Prediction models are used to forecast the protease-specific substrate cleavage; therefore, both peptides _695_YTMSLGAEN**S**VAYSNNS_711_ (S704 peptide) and _695_YTMSLGAEN**L**VAYSNNS_711_ (L704 peptide), including the amino acid substitution S704L, which might change the proteolytic digest by proteases, were assessed for cleavage prediction approach for NE (ExPASy (https://web.expasy.org/peptide_cutter/, accessed on 5 July 2022). As a result, NE hypothetically digested both peptides in the same manner, where the cleavage sites of the peptides occurred between _701_AE_702_, _705_VA_706_, and _706_AY_707_ (Figure 1 and Figure S1). In order to evaluate these data, S704 and L704 peptides were synthesized, treated with NE, CatG, or PR3, and the digestion pattern was analyzed by HPLC and MALDI-TOF-MS as well as high-resolution ESI-QTOF-HRMS. Strikingly, NE catalyzed the hydrolysis of S704 and L704 peptides exclusively at _705_VA_706_ (Figure 2 and Figures S2–S8). CatG hydrolyzed the peptide bond at _697_MS_698_, _699_LG_700_, and _707_YS_708_, which were identified for both peptides. PR3 digested the S704 and L704 peptides in the same manner as shown for NE; however, additional cleavage sites were detected between _704_SV_705_ for the S704 peptide that was eliminated in the L704 peptide. In contrast, a novel cleavage site was located between _701_AE_702_, which was most likely controlled by the P3′ position by the amino acid exchange from S to L.

The specific cleavage site of proteases within the S protein provides a deeper understanding of the mechanism of antigen processing. Thereby, the prediction tool of protease-controlled proteolysis supports such an insight and might lead to novel therapeutic targets. In accordance with the data obtained by the computational approach, three positions (_701_AE_702_, _705_VA_706_, and _706_AY_707_) are sufficient for NE to cleave the S704 and L704 peptides, which is contradictory to our findings where NE processed these peptides only at _705_VA_706_. In this regard, the in silico analysis has its limitations.

In general, the hydrolysis of the peptide bond is influenced by glycans and can regulate substrate binding and turnover. While N-linked glycans are covalently bound to defined asparagine residues (N), O-linked glycans are attached to characteristic serine/threonine residues (S/T) [15]. N709, N717, and N801 are such glycosylation locations downstream of the S2 subunit [16] and have the potency to change the proteolytic capacity to nearby residues. However, our short peptides cannot be applied to address the impact of glycosylation on proteolytic digestion.

The extracellular processing of the S2 subunit by neutrophil-derived serine proteases might have an impact on further intracellular antigen processing, binding to major histocompatibility complex I (MHC I, recognized by CD8^+^ T cells) and MHC II (monitored by CD4^+^ T cells), and the presentation of S2-derived peptides for T cell inspection. Recently, it was demonstrated that mutations in the S protein of SARS-CoV-2 mainly reduced antigenic peptide binding to several MHC II alleles suggesting immune evasion [17]. In the case of the S704L mutation and “preprocessing” of the S2 by PR3, a different set of antigenic peptides might be generated intracellularly, possibly impairing or varying the peptide binding properties to MHC I and II molecules, which can alter a T cell-mediated immune response.

## 3. Materials and Methods

### 3.1. Analysis of Peptides In Silico

FASTA format sequences of the S protein peptides SARS-CoV-2 (Wuhan, WIV04, UniProtKB: P59594, https://www.uniprot.org, accessed on 5 July 2022) _695_YTMSLGAEN**S**VAYSNNS_711_ or including the amino acid substitution S704L _695_YTMSLGAEN**L**VAYSNNS_711_. Both peptides were assessed for potential cleavage sites for human NE (EC 3.4.21.37) using in silico analysis of the program ExPASy Peptide Cutter (https://web.expasy.org/peptide_cutter/, accessed on 5 July 2022).

### 3.2. Peptide Digestion In Vitro and Analysis

_695_YTMSLGAEN**S**VAYSNNS_711_ and _695_YTMSLGAEN**L**VAYSNNS_711_ were synthesized (EMC Microcollections GmbH, Tübingen, Germany), purified (reversed-phase high-performance liquid chromatography, HPLC, C18 250 × 8 column, Dr. Maisch GmbH, Ammerbuch, Germany), and the exact mass was determined by mass spectrometry (matrix-assisted laser desorption/ionization-time-of-flight mass spectrometry, MALDI-TOF-MS, Autoflex, Bruker Daltonics, Bremen, Germany) by applying the matrix 2,5-Dihydroxybenzoic acid (DHB) (Bruker Daltonics, Bremen, Germany). Afterwards, the peptides were lyophilized and dissolved with PBS (pH 7) to a final concentration of 10 mg/mL.

Peptides (200 μg/mL) were incubated with human CatG (4 μg/mL, neutrophil-derived CatG, PN: 16-14-030107, Lot No. CG 2017-01, Athens Research and Technology, Athens, GA, USA), human NE (4 μg/mL, neutrophil-derived human NE, PN: 16-14-051200, Lot No. EH 2020-03, Athens Research and Technology, Athens, GA, USA), or 4 μg/mL PR3 (Athens Research and Technology, Athens, GA, USA) in PBS (pH 7.4). After an incubation time of 2 h at 37 °C [18], the digestion pattern was resolved by HPLC (intelligent Pump L-6200, Merck-Hitachi, Darmstadt, Germany), which was connected to a C18 column (Reprosil 100, 250 × 2 mm, 5 μm particle diameter, Dr. Maisch GmbH, Ammerbuch, Germany), and a linear acetonitrile gradient. The peptides were detected at 214 nm (UV Vis detector L-4200, Merck-Hitachi, Darmstadt, Germany) with a Chromato-Integrator (D-2500, Merck-Hitachi, Darmstadt, Germany). Subsequently, the collected samples were analyzed by MALDI-TOF-MS (Autoflex, Bruker Daltonics, Bremen, Germany) and the ExPASy FindPept tool (Expasy 3.0, Swiss Institute of Bioinformatics, Lausanne, Switzerland, https://web.expasy.org/findpept/, [19], accessed on 8 July 2022).

### 3.3. HPLC Electrospray Ionization Quadrupole Time-of-Flight High-Resolution Mass Spectrometry (HPLC-ESI-QTOF-HRMS)

Mass spectra were recorded on an HPLC-UV-HR mass spectrometer (MaXis4G with Performance Upgrade kit with ESI-Interface, Bruker Daltonics, Bremen, Germany). Samples were diluted with LC-MS-methanol (1:1), centrifuged (20,238 relative centrifugal force), and applied to a Dionex Ultimate 3000 HPLC system (Thermo Fisher Scientific, Waltham, MA, USA), which is coupled to the MaXis 4G ESI-QTOF mass spectrometer (Bruker Daltonics, Bremen, Germany). The ESI source was operated at a nebulizer pressure of 2.0 bar, and dry gas was set to 8.0 L⋅min^−1^ at 200 °C. MS/MS spectra were recorded in auto-MS/MS mode with collision energy stepping enabled. Sodium formate was used as an internal calibrant in each analysis. The routine gradient was 90% MilliQ deionized H_2_O with 0.1% formic acid and 10% methanol with 0.06% formic acid to 100% methanol with 0.06% formic acid in 20 min with a flow rate of 0.3 mL/min on a Nucleoshell**^®^** EC RP-C18 (150 × 2 mm, 2.7 μm, Macherey-Nagel, Düren, Germany).

## 4. Conclusions

The generation of a different set of antigenic peptides being loaded to MHC molecules, due to the different antigen processing capacities of PR3 based on the novel S704L mutation, might not be recognized by memory T cells. Hypothetically, this could be an additional reason for immune evasion and the impaired immune response of vaccinated individuals.

## Data Availability

Supplementary section S1 to S7.

## Supplementary Materials

The following supporting information can be downloaded at: https://www.mdpi.com/article/10.3390/molecules27185817/s1, Figure S1: Primary data from the “ExPASy peptide cutter”; Figure S2: HPLC data for peptide digest with NE; Figure S3: HPLC data for peptide digest with CatG; Figure S4: HPLC data for peptide digest with PR3; Figure S5: MALDI-TOF-MS data summary; Figure S6: Peptide digest with NE, high-resolution MS/MS spectra from peptides.; Figure S7: Peptide digest with CatG, high-resolution MS/MS spectra from peptides.; Figure S8: Peptide digest with PR3, high-resolution MS/MS spectra from peptides.

## Sample Availability

Samples of the peptides are available from the authors.
