# Supplementary material for: Variation of Proteolytic Cleavage Sites towards the N-Terminal End of the S2 Subunit of the Novel SARS-CoV-2 Omicron Sublineage BA.2.12.1
Source: Molecules. 2022 Sep 8;27(18):5817. doi: 10.3390/molecules27185817 (PMC9506045; doi:10.3390/molecules27185817)

Supplementary data S1:

```
            Elast
           Elast|
       Elast   ||

           |   ||
     YTMSLGAENSVAYSNNS
 1   ---------+-------   17


            Elast
           Elast|
       Elast   ||

           |   ||
     YTMSLGAENLVAYSNNS
 1   ---------+-------   17
```

Figure S1: Primary data from the "ExPASy peptide cutter". NE cleavage sites prediction of $_{695}$YTMSLGAEN**S**VAYSNNS$_{711}$ (S704 peptide) and $_{695}$YTMSLGAEN**L**VAYSNNS$_{711}$ (L704 peptide) by applying "ExPASy peptide cutter". Elast = elastase.

Supplementary data S2:

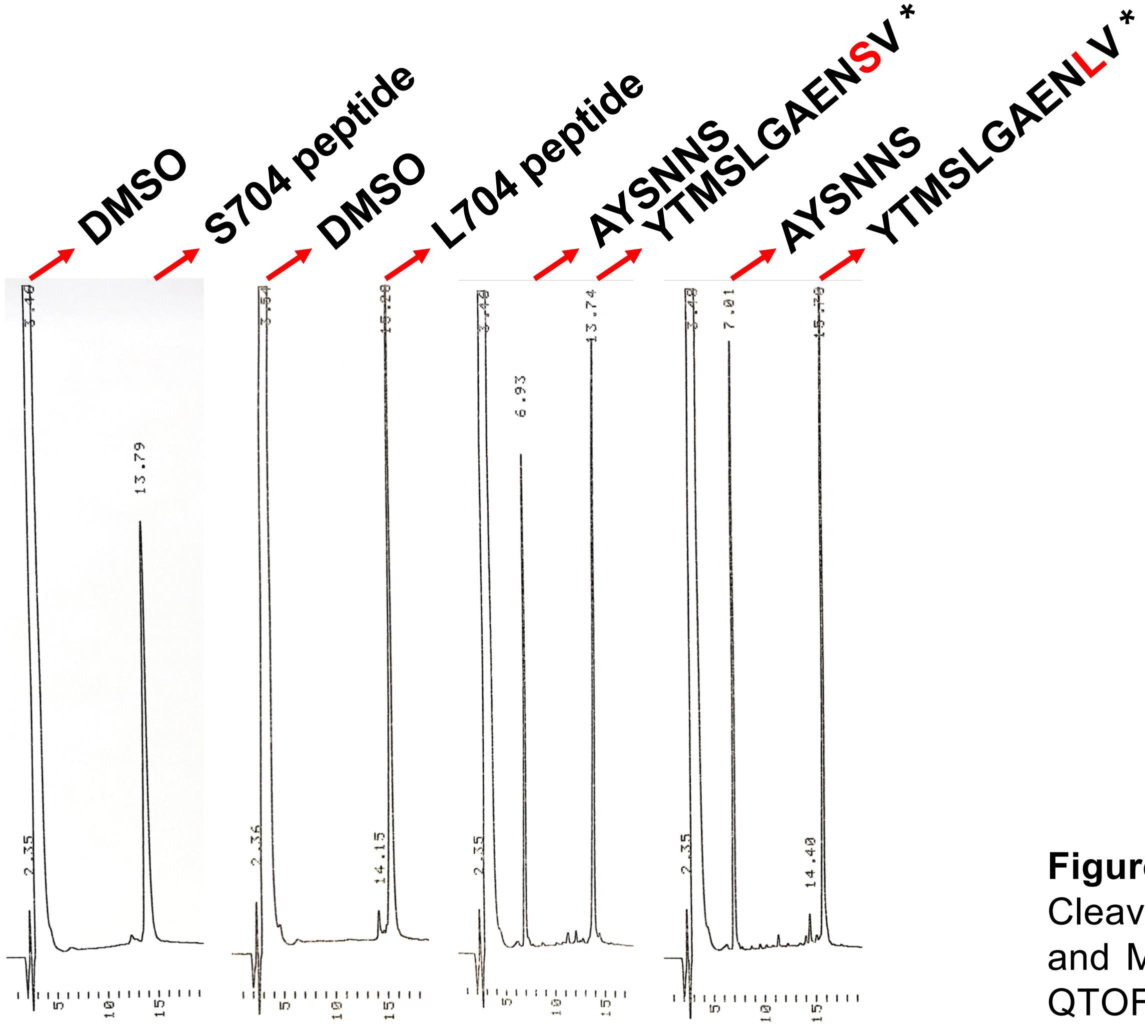

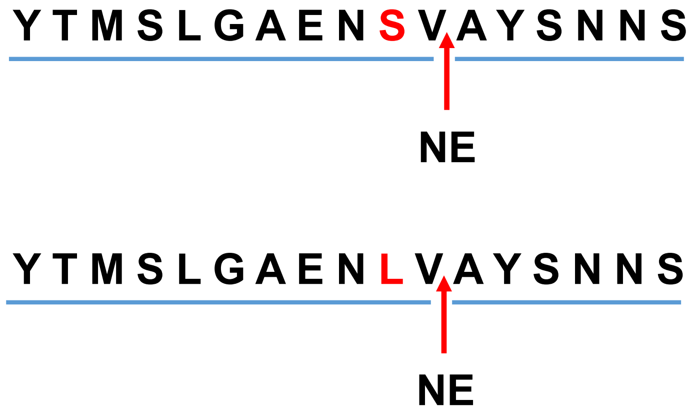

Figure S2: Primary HPLC data for peptide digest. Cleavage sites for NE were determined by HPLC and MALDI-TOF-MS. * Confirmed by HPLC-ESI-QTOF-HRMS.

Supplementary data S3:

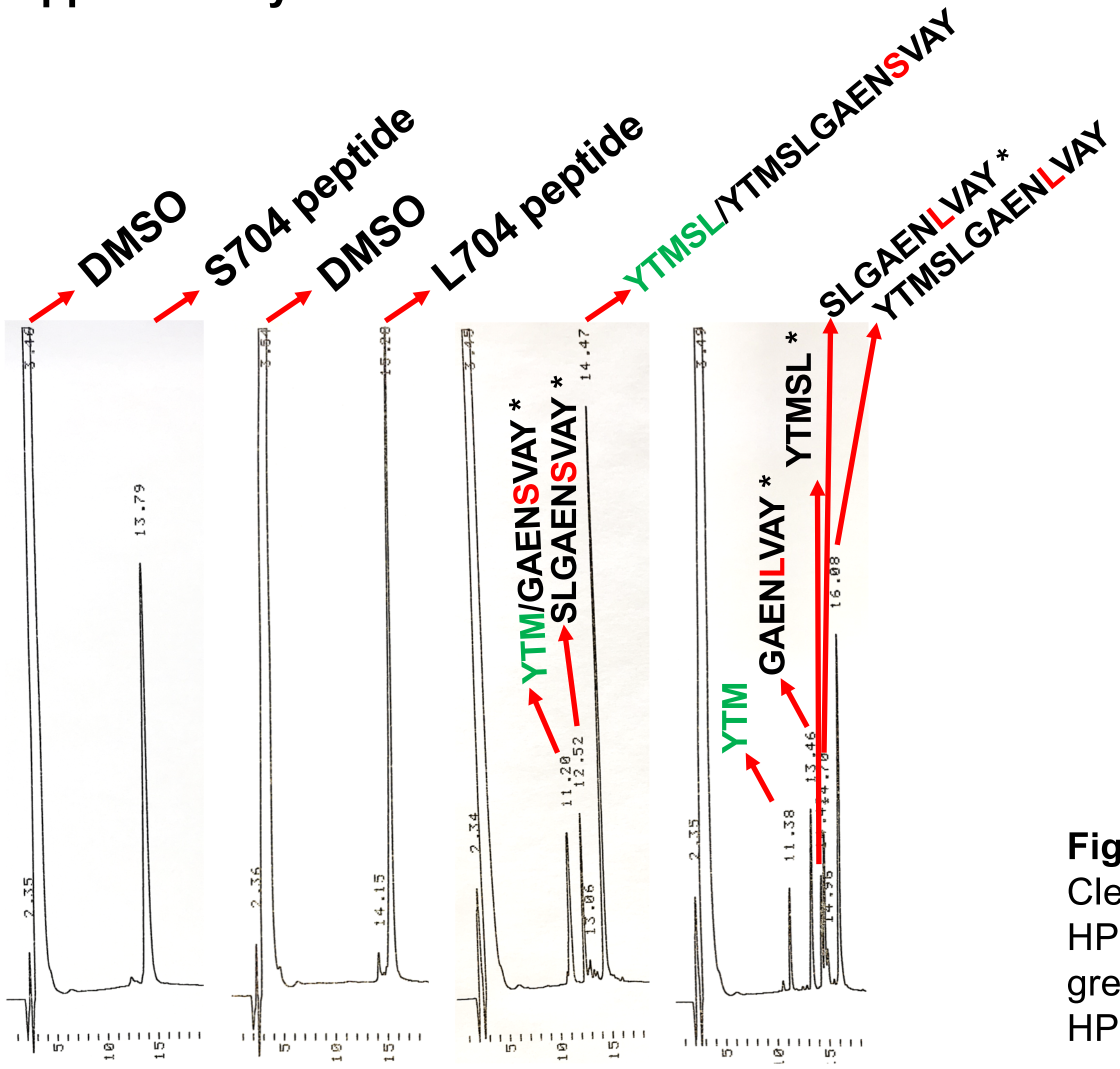

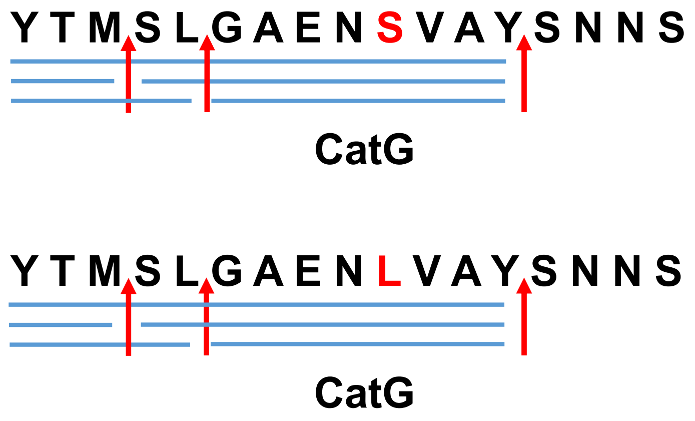

Figure S3: Primary HPLC data for peptide digest. Cleavage sites for CatG were determined by HPLC and MALDI-TOF-MS. Amino acids in green, HPLC-ESI-TOF-HRMS; * Confirmed by HPLC-ESI-QTOF-HRMS.

Supplementary data S4:

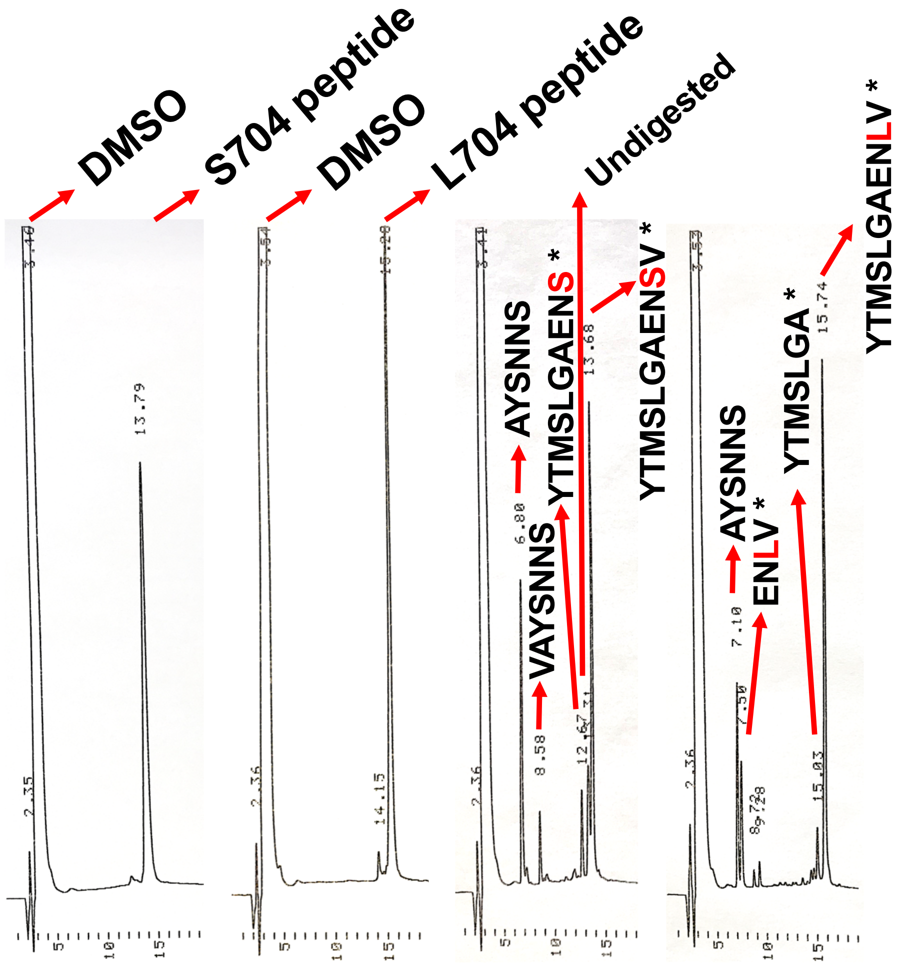

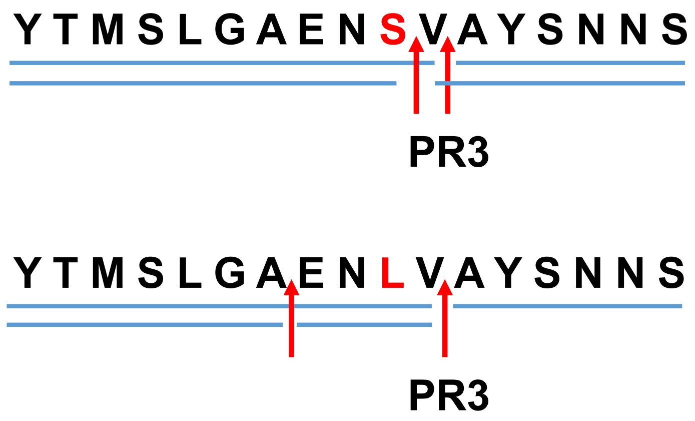

Figure S4: Primary HPLC data for peptide digest. Cleavage sites for PR3 were determined by HPLC and MALDI-TOF-MS. Amino acids in green, HPLC-ESI-TOF-HRMS; * Confirmed by HPLC-ESI-QTOF-HRMS.

Supplementary data S5:

S704 peptide			
**Protease**	**Mass [M+H]$^+$**	**Expected mass [M+H]$^+$**	**Peptide**
NE	655.45	655.27	AYSNNS
	1172.52	1171.53	YTMSLGAENSV
CatG	811.80	810.36	GAENSVAY
	1010.88	1010.48	SLGAENSVAY
	1405.27	1405.63	YTMSLGAENSVAY
PR3	655.43	655.27	AYSNNS
	754.46	754.34	VAYSNNS
	1072.95	1072.46	YTMSLGAENS
	1806.86	1807.78	undigested
	1171.84	1171.53	YTMSLGAENSV

L704 peptide			
**Protease**	**Mass [M+H]$^+$**	**Expected mass [M+H]$^+$**	**Peptide**
NE	655.47	655.27	AYSNNS
	1197.54	1197.58	YTMSLGAENLV
CatG	837.67	836.41	GAENLVAY
	615.61	614.23	YTMSL
	1036.81	1036.53	SLGAENLVAY
	1431.52	1431.68	YTMSLGAENLVAY
PR3	654.01	655.27	AYSNNS
	474.31	474.26	ENLV
	741.41	742.34	YTMSLGA
	1197.75	1197.58	YTMSLGAENSV

Figure S5: Mass spectrometry data from MALDI-TOF-MS.

Supplementary data S6: Figure S6: Peptide digest with NE, high-resolution MS/MS-spectra from peptides.

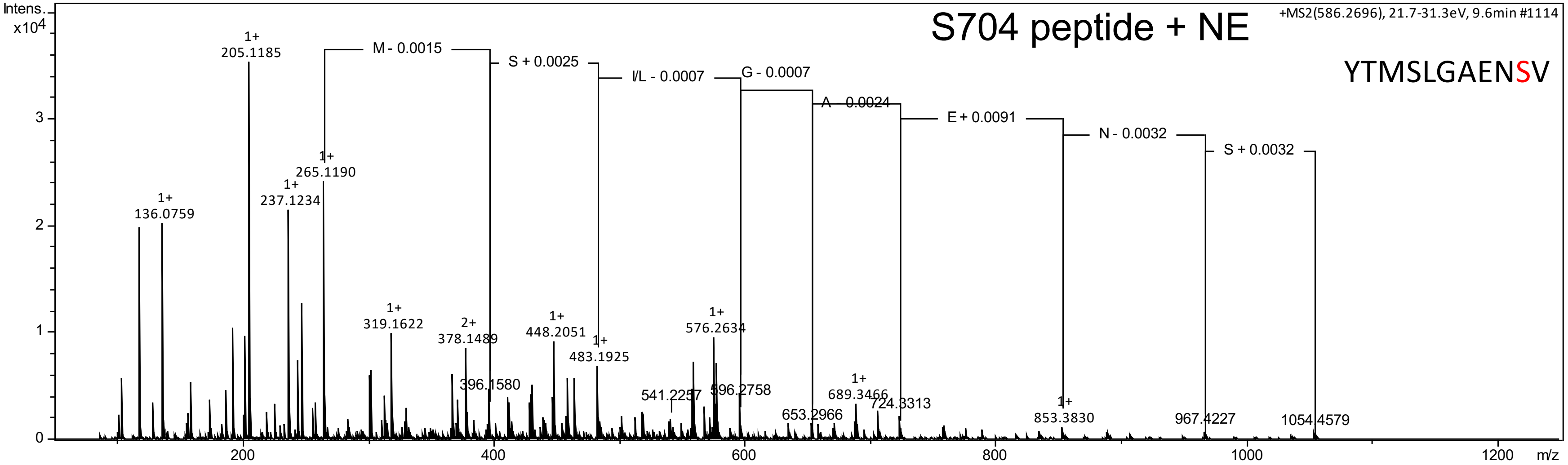

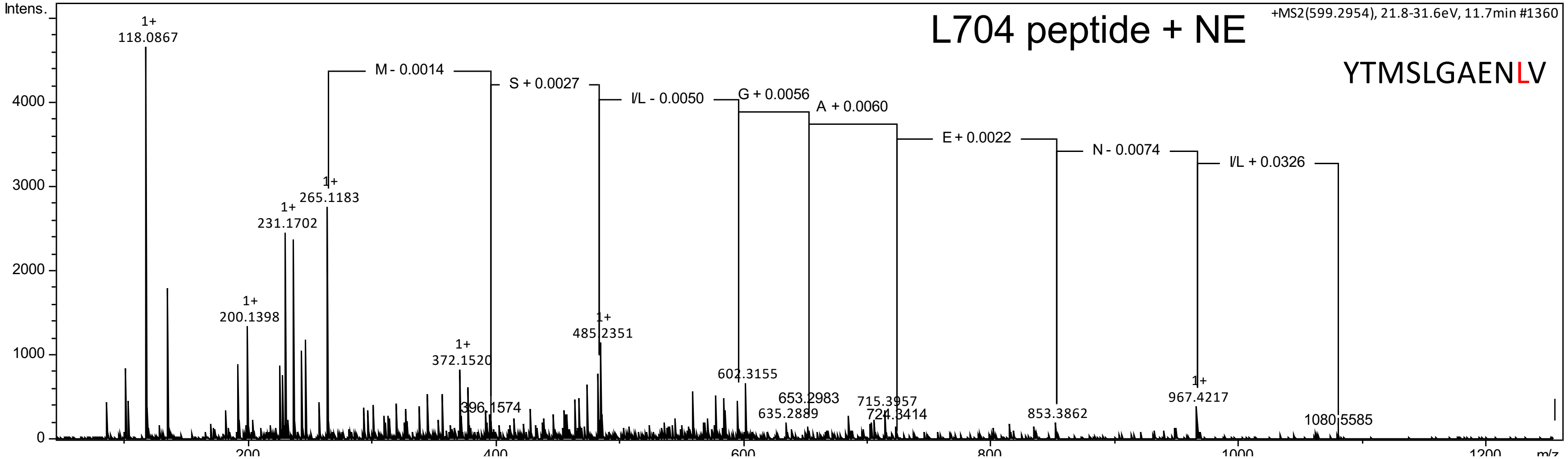

Supplementary data S7: Figure S7: Peptide digest with CatG, high-resolution MS/MS-spectra from peptides.

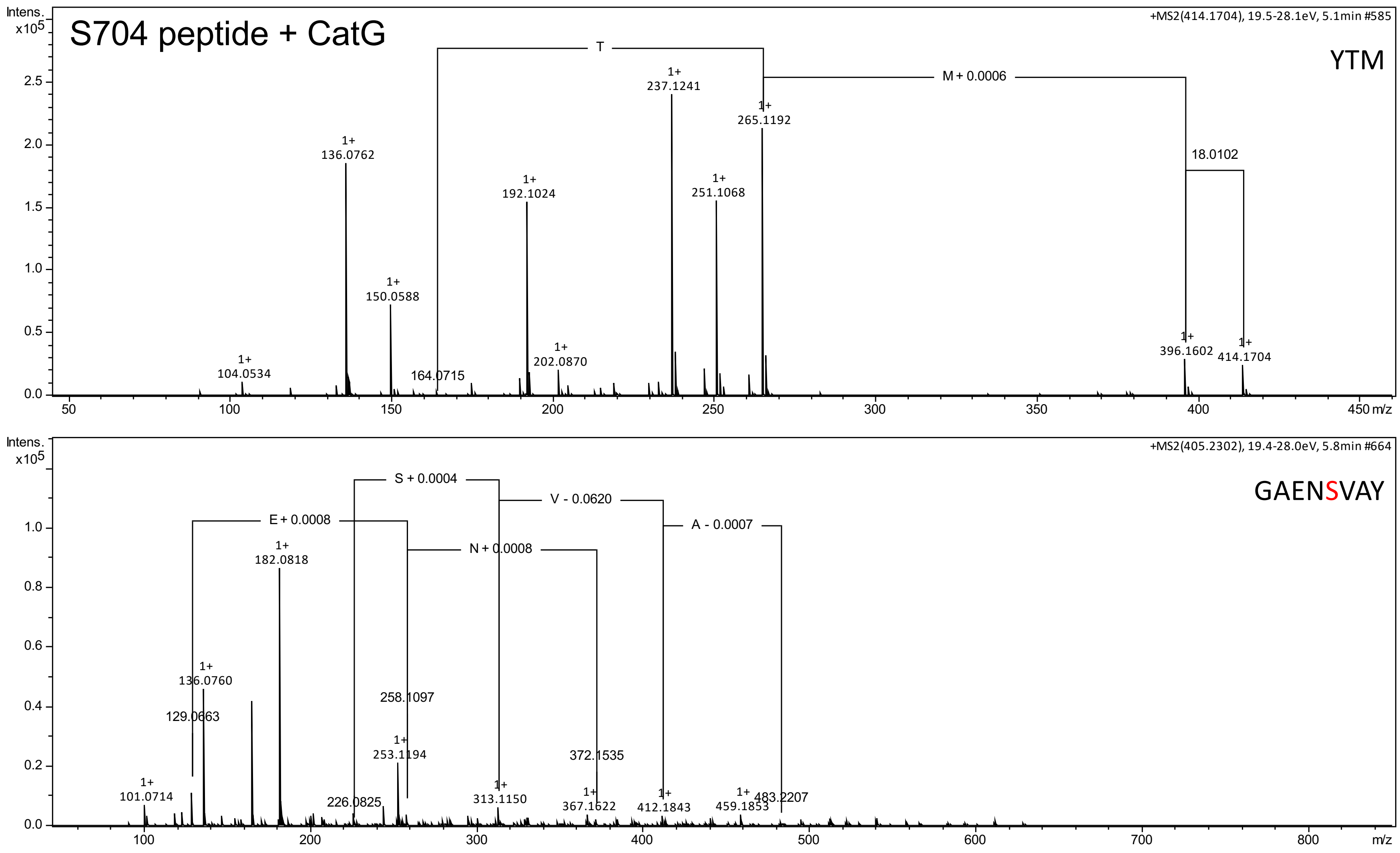

Supplementary data S7: Figure S7: Peptide digest with CatG, high-resolution MS/MS-spectra from peptides.

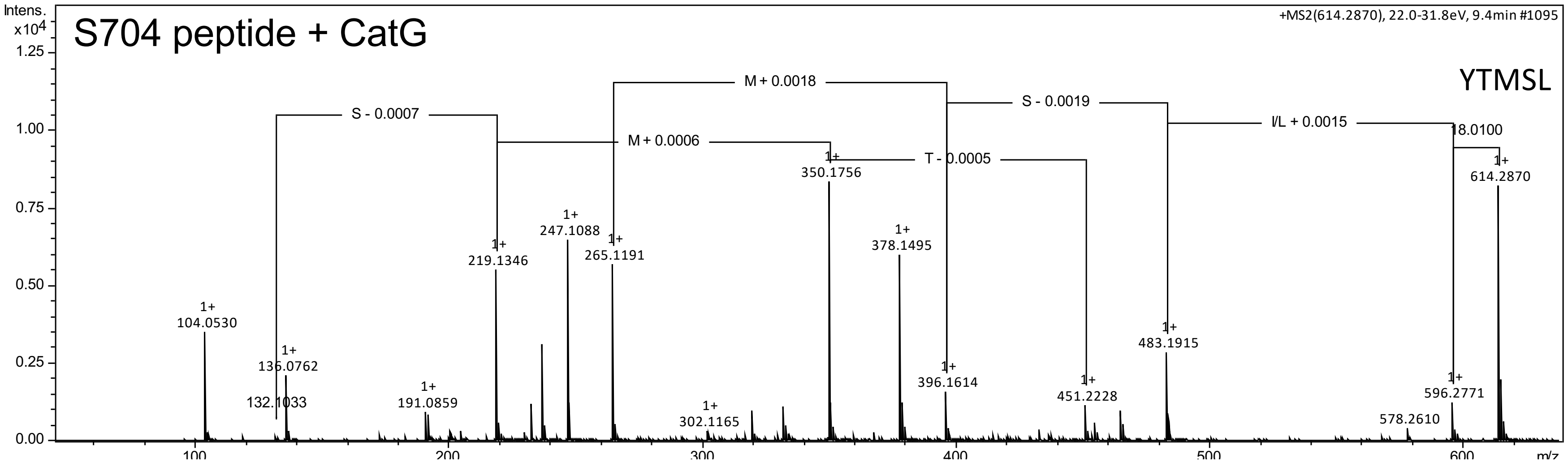

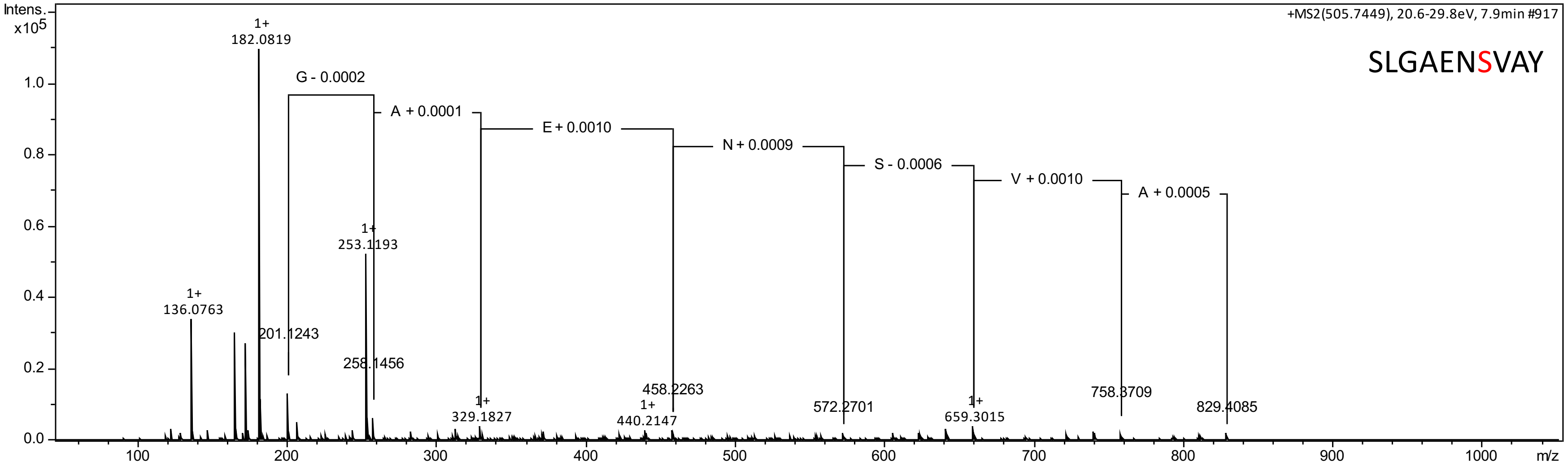

Supplementary data S7: Figure S7: Peptide digest with CatG, high-resolution MS/MS-spectra from peptides.

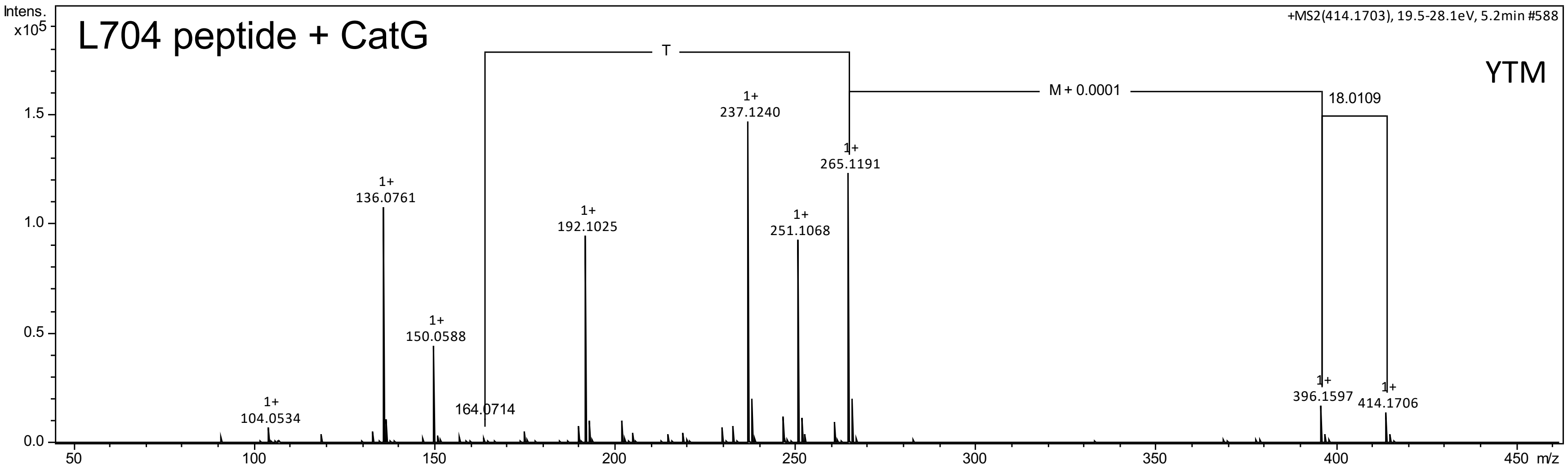

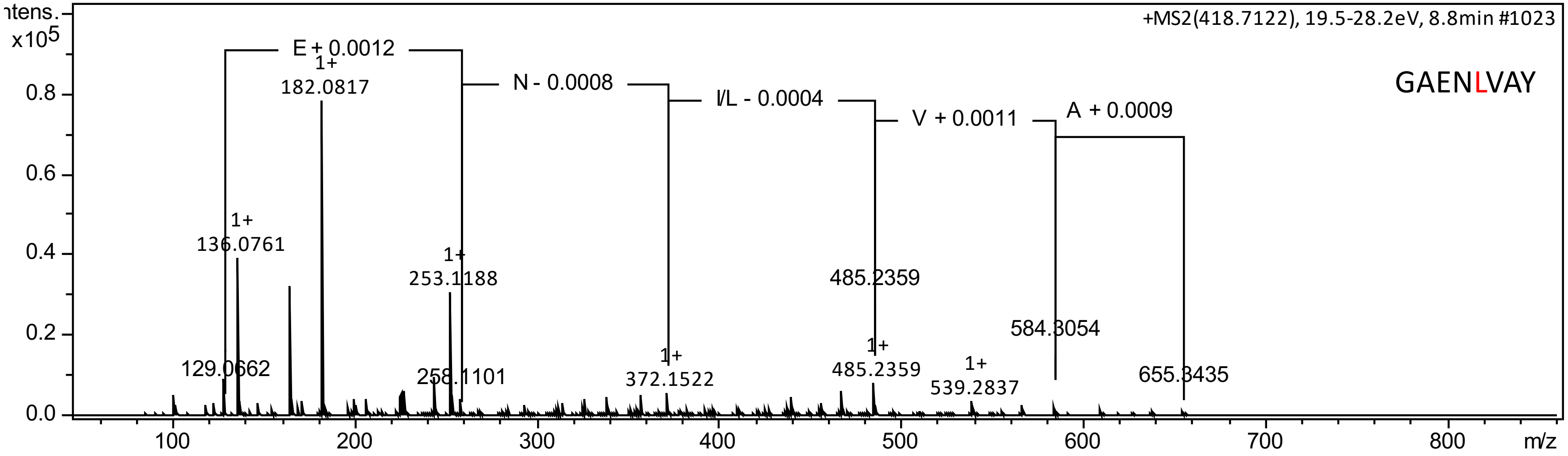

Supplementary data S7: Figure S8: Peptide digest with CatG, high-resolution MS/MS-spectra from peptides.

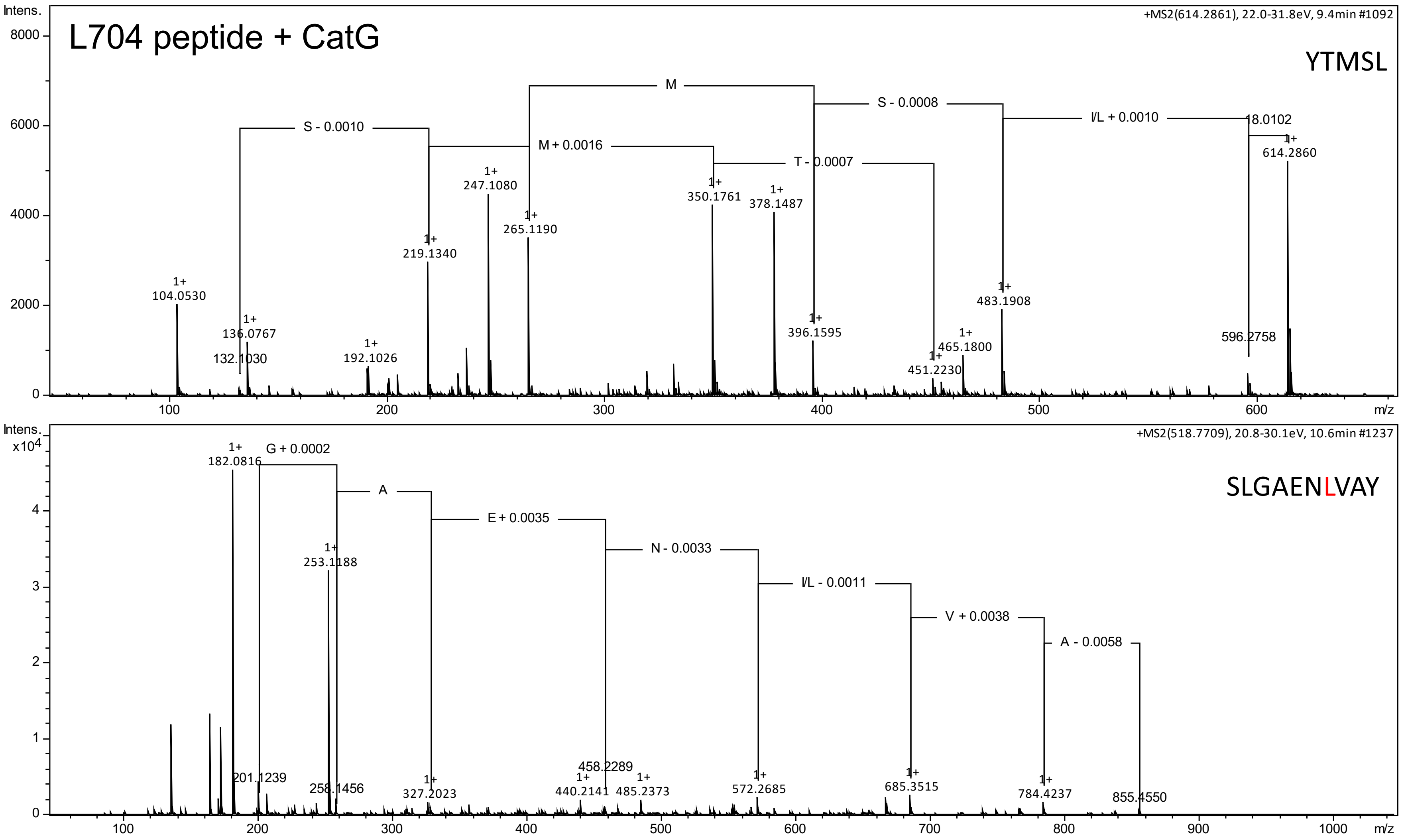

Supplementary data S8: Figure S8: Peptide digest with PR3, high-resolution MS/MS-spectra from peptides.

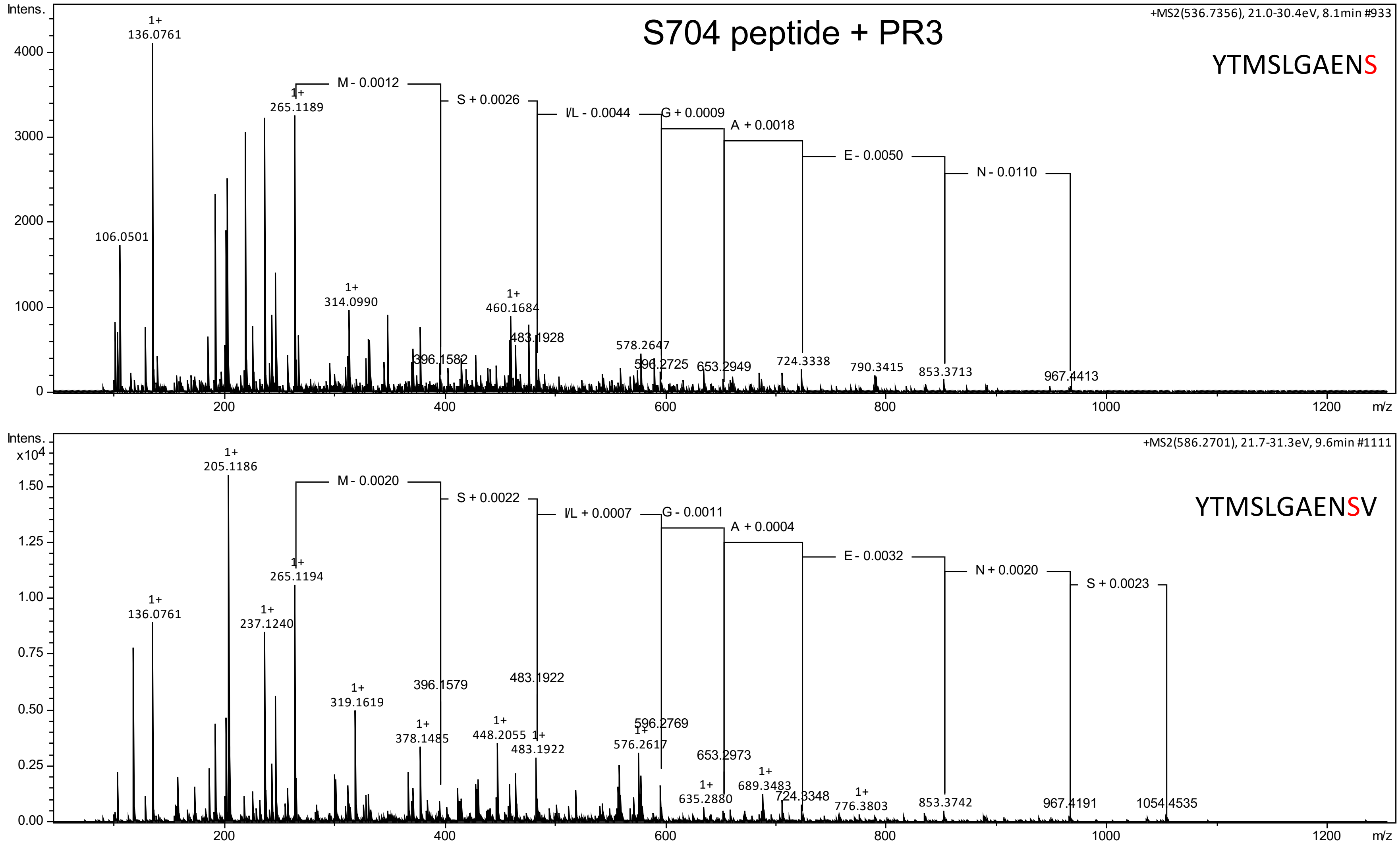

Supplementary data S8: Figure S8: Peptide digest with PR3, high-resolution MS/MS-spectra from peptides.

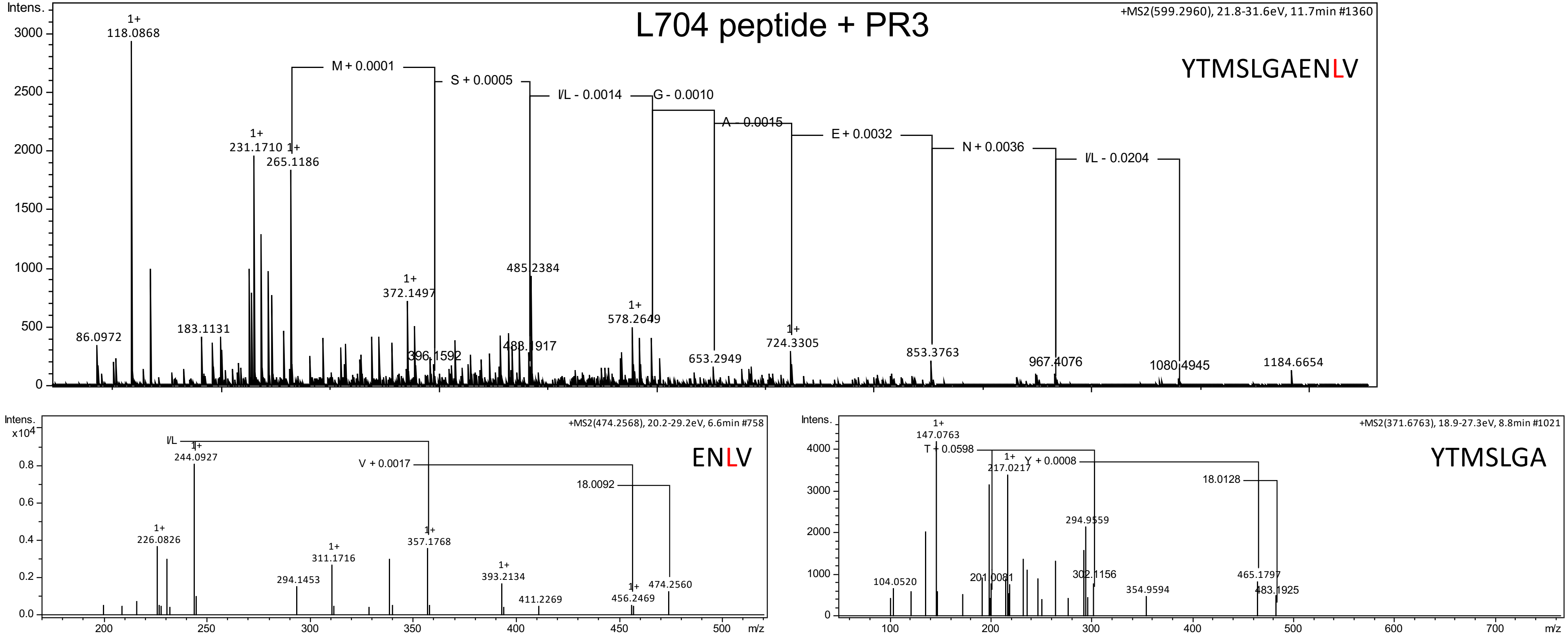

Supplement: Supplementary file 1 [file molecules-27-05817-s001.zip › molecules-1886773-supplementary.pdf]
